# Nicotinamide *N*‐methyltransferase promotes drug resistance in lung cancer, as revealed by nascent proteomic profiling

**DOI:** 10.1002/1878-0261.70097

**Published:** 2025-07-18

**Authors:** Zhanwu Hou, Zhen Wang, Fei Yang, Xiao Han, Lei Li, Huadong Liu

**Affiliations:** ^1^ Ministry of Education (MoE) Key Laboratory of Biomedical Information Engineering, School of Life Science and Technology Xi'an Jiaotong University China; ^2^ School of Life Science and Health University of Health and Rehabilitation Sciences Qingdao China

**Keywords:** cancer drug resistance, EGFR inhibitor, nascent proteome, NNMT, phosphorylation

## Abstract

Kinase inhibitors have achieved great success in targeted cancer therapy, yet the evident limitations in their effectiveness persist due to therapeutic resistance. To gain insight into the molecular mechanisms and thwart resistance, we profiled the time‐resolved nascent protein perturbations in response to drug therapy using metabolic labeling and facilitated the identification of 2238 proteins via liquid chromatography tandem mass spectrometry (LC–MS/MS). Among these, 51 proteins exhibited upregulation, whereas 105 proteins showed downregulation following a 24‐h drug treatment. Clustering analysis revealed that the differential proteins were mainly enriched in metabolic‐related pathways. Combined with changes in whole‐protein levels, we noticed significant fluctuations in the metabolism‐related protein nicotinamide *N*‐methyltransferase (NNMT). Additionally, NNMT overexpression diminished drug effectiveness, whereas its inhibition enhanced therapeutic efficacy. An increase in NNMT was also found in drug‐resistant cells, and the NNMT inhibitor JBSNF‐000088 inhibited the proliferation of resistant cells. Subsequent phosphoproteomic analysis indicated that the effects of NNMT overexpression on transcription factors, proteins involved in the Rho GTPases cycle, and cell‐cycle‐related proteins may be related to tumor resistance. In summary, our study provides unique insights into nascent protein perturbations during the initial stages of drug therapy and identified NNMT as a promising target for delaying and overcoming therapeutic resistance.

## Graphical abstract

AZD9291 has shown promise in targeted cancer therapy but is limited by resistance. In this study, we employed metabolic labeling and LC–MS/MS to profile time‐resolved nascent protein perturbations, allowing dynamic tracking of drug‐responsive proteins. We demonstrated that increased NNMT expression is associated with drug resistance, highlighting NNMT as a potential target to overcome resistance.

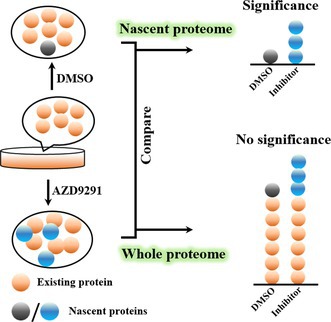

**Abbreviations**
AHA: azidohomoalanine
EGFR: epidermal growth factor receptor
GO: gene ontology
JBSNF: JBSNF‐000088
LC–MS/MS: liquid chromatography tandem mass spectrometry
NNMT: nicotinamide *N*‐methyltransferase
NNMT‐OE: NNMT overexpression
PCA: principal component analysis
PRM: parallel reaction monitoring
Rc: rate of change
TAD‐resin: terminal alkyne and disulfide functionalized agarose resin
WB: western blot

## 1 Introduction

Epidermal growth factor receptor (EGFR) mutations are pivotal drivers of signal network dysregulation in lung cancer, prompting the development of several EGFR inhibitors for targeted therapies, which have made a significant clinical breakthrough [1, 2]. Despite inducing profound initial responses that suppress cancer proliferation, targeted therapies often face immediate cellular responses that enable cell survival and eventual establishment of new homeostasis, leading to therapeutic resistance [3, 4]. Studies focusing on kinome reprogramming have unveiled diverse mechanisms fostering cancer cell survival and drug resistance, exemplified by the observed upregulation of AURKA following EGFR inhibition [5, 6]. In response, several therapeutic strategies have been devised to delay and overcome resistance [7]. Similarly, analyses of global proteomic immediate alterations upon inhibitor stimulation have elucidated underlying mechanisms promoting drug resistance [8, 9]. However, the presence of numerous existing proteins can attenuate the signaling of protein perturbations [10, 11]. Consequently, monitoring the immediate proteome response to stimuli at an early stage remains challenging [12].

In recent years, nascent protein metabolic labeling has emerged as a novel strategy for monitoring immediate proteome responses to stimuli [13]. Nascent proteome refers to the proteome consisting of newly synthesized proteins during a certain period [14]. Cells can adapt to stimulation by synthesizing new proteins and altering the ensemble of the proteome [15]. Consequently, measuring nascent proteome responses to inhibitors facilitates early interventions [16]. Specifically, labeling newly synthesized proteins using azidohomoalanine (AHA) enables spatiotemporal visualization of immediate nascent proteome changes after inhibitor treatment, aided by MS‐based analysis [17, 18]. It is crucial for enhancing therapeutic efficiency and mitigating therapeutic resistance.

AZD9291 is a third‐generation irreversible EGFR inhibitor, which provides clinical benefit for patients with resistance to the first‐ and second‐generation EGFR inhibitors [19]. However, cancer drug resistance is still inevitable, leading patients to relapse [20]. An understanding of alterations in global proteome and phosphoproteome revealed multiple mechanisms that promote cancer cell survival and AZD9291 resistance, including β‐catenin activation [20], PTK7 overexpression [21] and PI3K/AKT activation [22]. However, immediate alteration in nascent proteome after AZD9291 treatment remains unexplored. It is a critical step that enrichment of nascent proteins from existing proteins for nascent proteome analysis [23]. Previously, we have used a terminal alkyne and disulfide functionalized agarose resin (TAD‐resin) to enrich nascent proteins specifically [24]. Employing TAD‐resin‐based quantitative mass spectrometry, we characterized immediate alterations in nascent proteome under AZD9291 treatment and identified 2238 nascent proteins, with 156 significantly regulated after 24‐h treatment. Subsequently, whole‐protein analysis via parallel reaction monitoring (PRM) revealed high NNMT expression under AZD9291 treatment, and NNMT overexpression diminished drug effectiveness, while inhibition enhanced therapeutic efficacy. Furthermore, systematic phosphoproteomic analysis revealed the effect of NNMT on phosphorylation signals and suggested that NNMT may play a regulatory role by influencing transcription factors, such as MAPK1/3 and STAT3, not only through epigenetic regulation. This study is the first analysis of nascent proteome in cancer cells under kinase inhibitor treatment, pointing out that NNMT is a potential factor to promote cell survival, and is expected to be a potential target to overcome drug resistance.

## 2 Materials and methods

### 2.1 Chemicals and materials

Bicinchoninic acid kit (23227) and formic acid (28905) were purchased from Thermo (Horsham, UK). Protease inhibitor cocktail (535140) and nitrocellulose filter membranes (HATF00010) were obtained from Millipore (Gillingham, UK). Medium (31800022) and fetal bovine serum (FBS) (A5256701) were purchased from Gibco (California, USA). AZD9291 (HY‐15772A) and JBSNF‐000088 (HY‐112584) were obtained from MedChemExpress (Monmouth Junction, NJ, USA). Crystal violet (A600331) and MTT (A600799) were purchased from Sangon Biotech (Shanghai, China). Trypsin (V5111) was purchased from Promega (Madison, WI, USA), and acetonitrile (100029) was purchased from Merck (Darmstadt, Germany). Antibodies against phospho‐EGFR (Y1068) (3777), pY‐100 (9411), NNMT (33361), and β‐actin (4967) were purchased from Cell Signaling Technology (Leiden, Netherlands); antibody against biotin (SA00001‐0) was purchased from Proteintech (Hubei, China). Water used in the experiments was derived from a Milli‐Q system (Millipore).

### 2.2 Cell culture

H1975 (RRID:CVCL_1511) and HEK293T (RRID:CVCL_0063) cell lines were obtained from Cell Bank of Shanghai Institutes for Biological Sciences, Chinese Academy of Sciences (Shanghai, China). H1975 and HEK293T were authenticated using short tandem repeat analysis within 3 years. Authentications were confirmed by a more than 80% match compared with the reference short tandem repeat profiles from the ATCC cell bank. The cells also underwent routine mycoplasma testing by PCR to exclude mycoplasma contamination. H1975 cells were cultured in RPMI 1640 medium supplemented with FBS, penicillin, and streptomycin. HEK293T cells were cultured in DMEM medium. All cells were maintained at 37 °C in a 5% CO_2_ humidified environment.

### 2.3 Cell viability assay

Cells were seeded into 96‐well microplates at a suitable density and incubated at 37 °C and 5% CO_2_ overnight. Then, the cells were treated with inhibitors for the indicated times. The relative cell viability was determined by MTT assay. Briefly, 20 μL of MTT solution (0.5 mg·mL^−1^) was added to each well following the time schedule, and the cells were incubated at 37 °C for 4 h. Then, MTT‐supplemented media were replaced with 150 μL of DMSO per well, and plates were gently shaken. The absorbance was measured at 570 nm by a Molecular Devices Flexstation 3 unit.

### 2.4 Clonogenic growth assay

Colony outgrowth assays were performed using crystal violet staining based on a previous method [25]. Briefly, cells were seeded into 6‐well plates at a low density of 1000 cells per well and cultured in a humidified incubator at 37 °C. Appropriate drugs were added after an additional 24 h and were changed every 3 days. After being exposed to drugs for 2 weeks, the colonies were fixed in 4% formaldehyde, stained with 0.1% crystal violet, and photographed after washing off excess crystal violet. Then, colonies were destained with 10% acetic acid for 15 min on a rocker and measured at 590 nm with a Molecular Devices Flexstation 3 unit.

### 2.5 Western blot assay

The whole‐cell proteins were extracted by western lysis buffer (P0013, Beyotime, Shanghai, China) and quantified by the bicinchoninic acid assay. Equal amounts of protein were separated and transferred onto nitrocellulose filter membranes. The membranes were blocked at room temperature for 1 h and then incubated overnight with the indicated primary antibodies at 4 °C. Following washes, membranes were incubated with secondary antibodies, and signals were visualized by a Chem imaging system (CliNX Science, Shanghai, China).

### 2.6 RNA extraction, reverse transcription, and qRT‐PCR

Total RNA was extracted using RNAiso Plus reagent (9108Q, Takara, Shiga, Japan) and quantified using Nanodrop (DeNovix, Guangzhou, China). 1 μg of total RNA was subjected to reverse transcription using the Evo M‐MLV RT kit (AG11728, Accurate Biology, Hunan, China), and 10 ng cDNA was applied for qPCR with SYBR® Green (AG11701, Accurate Biology, Hunan, China). NNMT relative expression was compared by $2^{-\Delta \Delta C_{t}}$ method and β‐actin served as the control. The primers used were as follows: NNMT: forward, 5′‐GAGCAGAAGTTCTCCAGCCT‐3′, and reverse, 5′‐ACCATTCGATTGTGTAGCCA‐3′; β‐actin: forward, 5′‐TCCTGTGGCATCCACGAAACT‐3′, and reverse, 5′‐GAAGCATTTGCGGTGGACGAT‐3′.

### 2.7 Gene overexpression

The stable overexpression of human NNMT in cell lines was achieved by using lentivirus vectors. H1975 cells were infected with lentivirus and following a 2‐week selection to obtain NNMT overexpression (NNMT‐OE) cell lines.

### 2.8 Metabolic label and whole‐cell protein extract

AHA was used to label nascent proteins. Cells were first incubated in a methionine‐lacking serum‐free medium for 2 h and then in an AHA‐containing medium for 4 h. After labeling, cells were washed with PBS, harvested using lysis buffer, sonicated, and centrifuged to obtain whole‐cell proteins.

### 2.9 Nascent protein enrichment and trypsin digestion

The nascent proteins were enriched by TAD‐resin from whole‐cell proteins according to a previous procedure [24]. Enriched nascent proteins were digested with trypsin (1 : 50 w/w enzyme : protein ratio) at 37 °C for 16 h. The peptide products were separated in a home‐made reverse‐phase C18 column, dried in a speed vacuum, and resuspended in MS loading buffer for LC–MS/MS analysis.

### 2.10 Mass spectrometric analysis

The digested peptides were analyzed on a Q‐Exactive plus MS equipped with an UltiMate 3000 RSLCnano system, using a C18 reversed‐phase analytical column. Data were acquired in data‐dependent acquisition mode for eluted peptides and processed using maxquant for identification and label‐free quantification. Targeted data acquisition was performed using scheduled PRM mode, with data processed using Skyline for quantification.

### 2.11 Software and statistical analysis

Various analyses including heatmaps, volcano plots, principal component analysis (PCA), functional pathway analysis, and KEGG enrichment were performed using origin (OriginLab Corporation, Northampton, MA, USA), string (https://cn.string‐db.org), and cytoscape (San Diego, California, USA). graphpad prism 5 (GraphPad Software, lnc., San Diego, California, USA) was utilized for statistical analyses, with significance thresholds set at *P < 0.05, **P < 0.01, and ***P < 0.001.

## 3 Results

### 3.1 Nascent proteome labeling of AZD9291‐treated H1975 cells

**Fig. 1:**
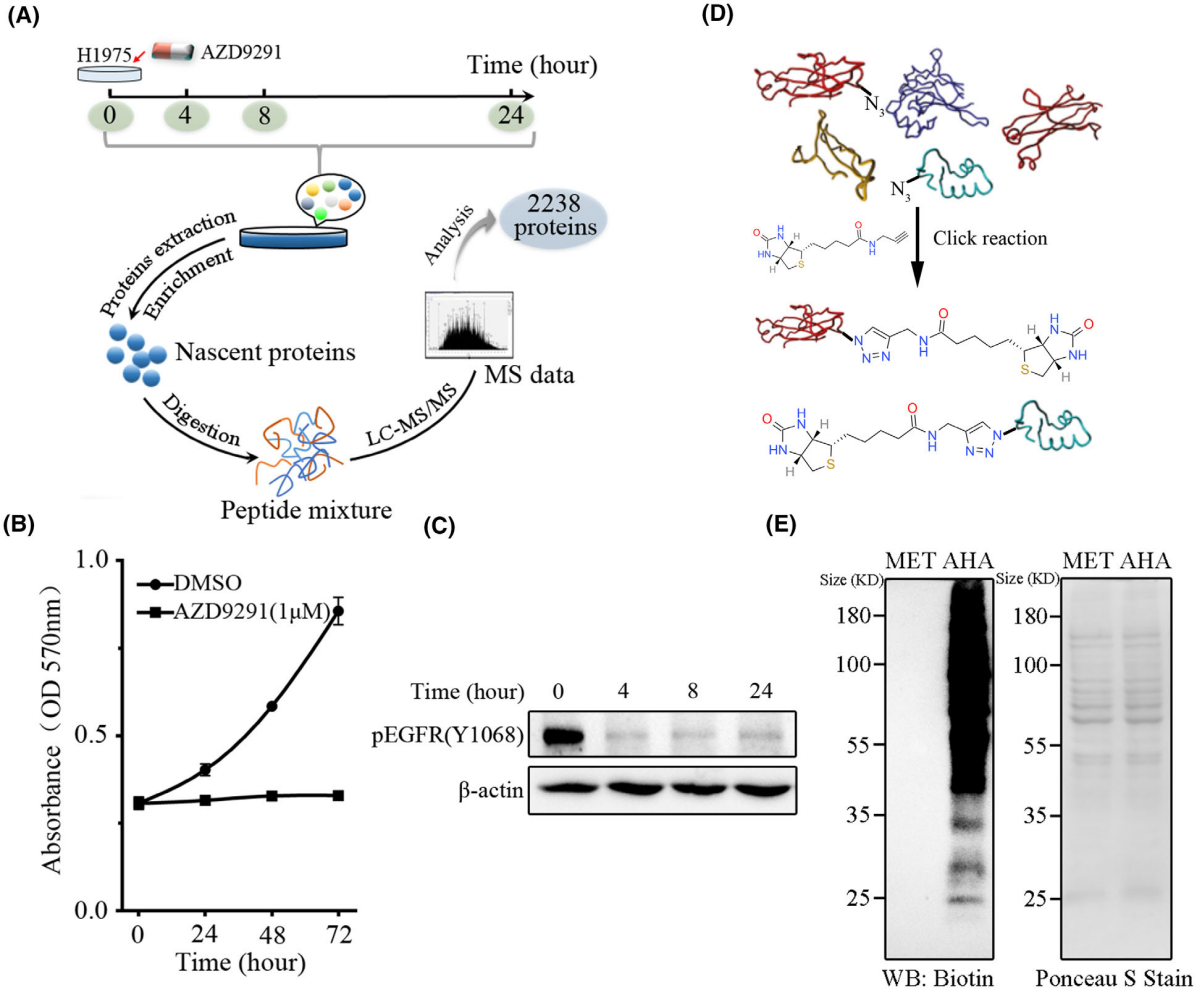
Metabolic labeling for studying the nascent proteome in H1975 cells. (A) Workflow for nascent protein labeling and identification in AZD9291‐treated H1975 cells. (B) Proliferation of H1975 cells after treatment with AZD9291 (1 μm) were assayed by MTT (*n* = 6). Error bars indicate standard deviation. (C) The phosphorylation of epidermal growth factor receptor (EGFR) after AZD9291 (1 μm) treatment (*n* = 3). (D) The nascent proteins containing azidohomoalanine (AHA) are biotinylated by click reaction. (E) Western blot (WB) was used for nascent proteome analysis (*n* = 3).

To identify and quantify the abnormal protein perturbations under the AZD9291 treatment, the nascent proteins were labeled by AHA, captured by TAD‐resin, and analyzed by LC–MS/MS (Fig. 1A). AZD9291 effectively inhibited the proliferation of H1975 cells harboring EGFR^L858R^ and EGFR^T790M^ dual‐mutations, which are resistant to first‐generation EGFR inhibitor gefitinib (Fig. 1B and Fig. S1A). Western blot (WB) analysis revealed a significant reduction in EGFR phosphorylation and overall intracellular tyrosine phosphorylation following AZD9291 treatment (Fig. 1C and Fig. S1B). Our previous study had shown that long‐term exposure of H1975 cells to AZD9291 promoted the development of drug resistance [25]. Understanding the immediate proteome changes in H1975 cells upon initial AZD9291 stimulation is crucial for uncovering the underlying mechanisms of resistance. Therefore, we employed AHA and alkyne‐biotin to label the nascent proteome of AZD9291‐treated H1975 cells, respectively (Fig. 1D). Finally, the nascent proteome was detected by WB using streptavidin‐HRP. As shown in Fig. 1E, although the ponceau S staining result showed a similar amount of proteins loaded, AHA cultured cells exhibited robust signals in comparison with control cells, which indicated that the nascent proteome was labeled and detected successfully. Then, AZD9291‐treated cells were labeled and detected using this strategy.

### 3.2 Identification and analysis of nascent proteome by LC–MS/MS

We used AHA to label the nascent proteome from H1975 cells exposed to AZD9291 for 4, 8, and 24 h, respectively. The whole proteins were reacted with alkyne‐biotin for WB analysis, and the result showed that the nascent proteome was labeled successfully. Disappointedly, there were no significant differences in WB results (Fig. S2A). Therefore, the nascent proteins were enriched by TAD‐resin for MS analysis, and the result showed that the nascent proteins were captured completely (Fig. S2B). Then, the enriched nascent proteins were subjected to trypsin digestion and LC–MS/MS analysis. Three separate biological replicates were performed for each set of experiments, and a total of 2238 proteins were identified. Among the identified proteins, we quantified 1433 proteins (Fig. S2C, Table S1).

**Fig. 2:**
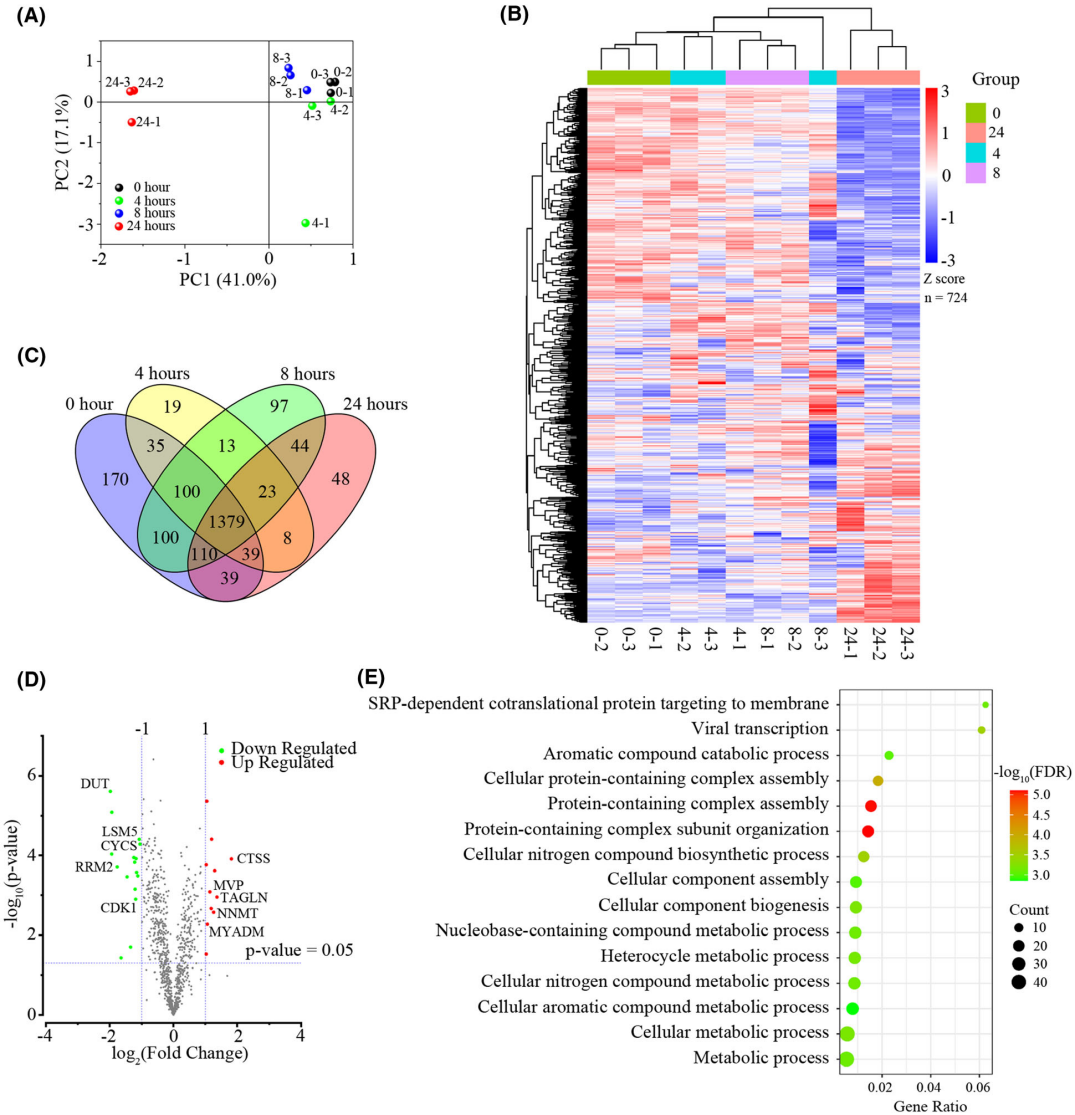
Nascent proteome analyzed by liquid chromatography tandem mass spectrometry (LC–MS/MS) after treatment with AZD9291 (1 μm) for the indicated time. (A) Principal component analysis (PCA) analysis of quantified nascent proteins, where numbers represent the duration of drug treatment (*n* = 3). (B) Heatmaps of hierarchical clustering in nascent protein levels. (C) Venn diagram showing the number of nascent proteins identified. (D) Volcano plots showing the nascent proteins changed after treatment with AZD9291 for 24 h (*n* = 3). (E) Gene ontology (GO) enrichment analysis of significantly changed nascent proteins after treatment with AZD9291 for 24 h.

PCA result revealed that the cells treated for less than 8 h exhibited similar nascent proteome profiles, and nascent proteome changes were distinctive after 24 h treatment (Fig. 2A). Then, we used label‐free quantification for z‐score normalization and clustering (Fig. 2B). As expected, the control group showed the same trend as the 4‐ and 8‐h groups but showed a significant difference from the 24‐h group, which was consistent with PCA results. Among the nascent proteins, 1379 proteins were present in all four groups, which were necessary for cell proliferation and survival (Fig. 2C). Moreover, volcanic map also showed few differentials in nascent proteins between the 0‐ and 4‐ or 8‐h (Fig. S2D,E). However, there was a significant difference in nascent proteome at 24 h compared to 0 h (Fig. 2D). After 24 h of AZD9291 stimulation, cell growth slowed down and the synthesis of some proteins such as DUT, RRM2, CDK1, and CYCS was also reduced. On the contrary, there were several proteins whose synthesis was increased after drug treatment, such as CTSS, TAGLN, NNMT, and MYADM (Fig. S2F). We performed gene ontology (GO) analysis, and the differential proteins were mainly enriched in metabolism‐related processing, indicating the metabolic alteration was an important pathway in the cellular response to AZD9291 (Fig. 2E).

### 3.3 Comparison of nascent proteome and whole proteome

**Fig. 3:**
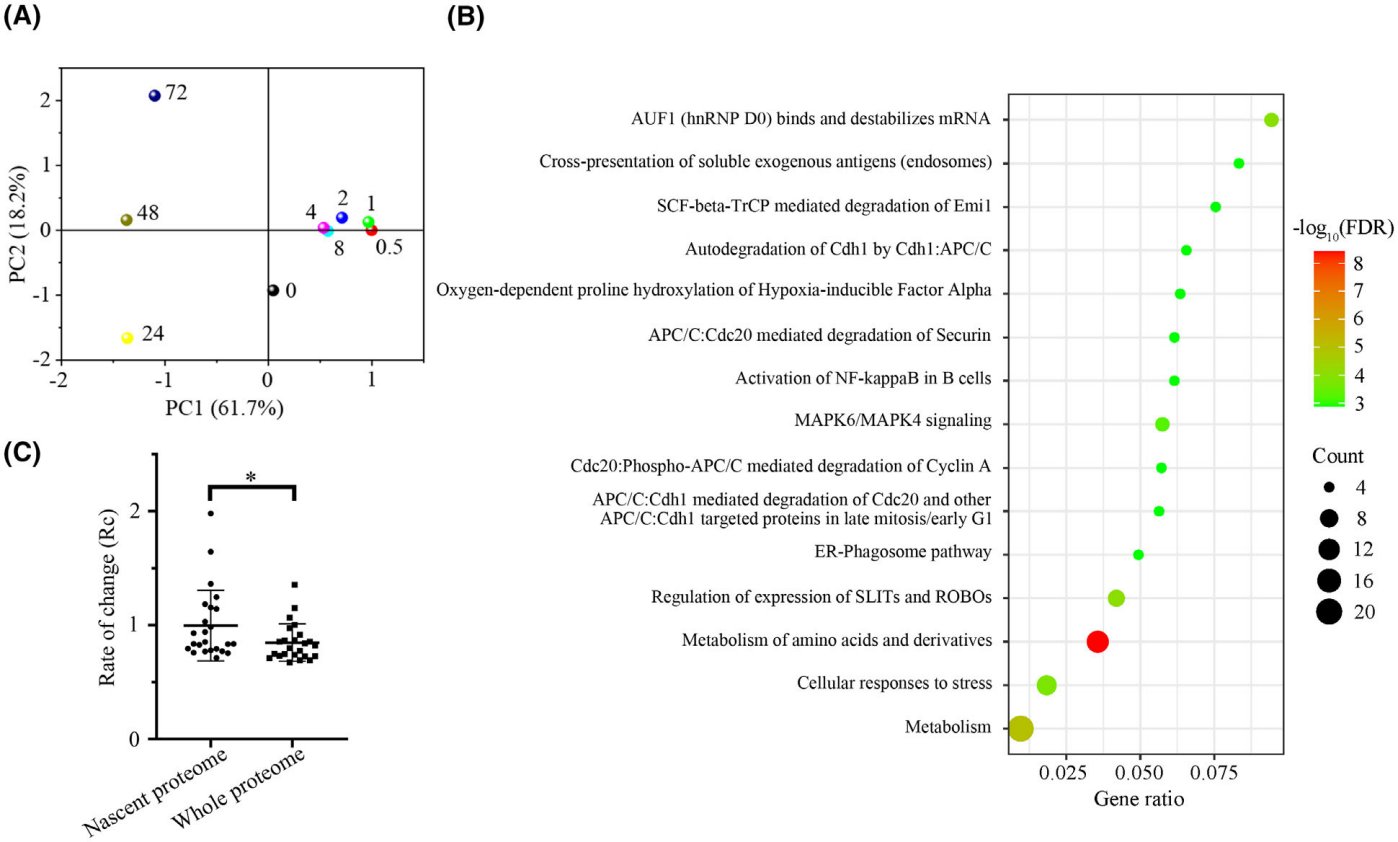
Whole proteome analyzed by parallel reaction monitoring (PRM) during the AZD9291 (1 μm) treatment. (A) PCA analysis of proteins during different stages of AZD9291 treatment, where numbers indicate the duration of drug treatment (*n* = 2). (B) Reactome analysis of the significantly changed proteins in whole‐protein level after treatment with AZD9291 for 72 h. (C) Comparison of rate of change (Rc) between nascent proteome and whole proteome (*n* = 2). Error bars depicting standard deviation. Significance determined by Student's *t*‐test. *P < 0.05.

Based on the nascent proteome data, an optimized scheduled PRM method was established to quantitatively analyze 515 unique peptides (Table S2). We then used this method to quantify whole‐protein alteration after AZD9291 treatment. Finally, 397 unique peptides were obtained with high‐quality spectra, which can be used to quantify the abundance of 158 proteins (Fig. S3, Table S3). PCA analysis showed that the whole‐protein changed little within 8 h of treatment, and the difference became more obvious with the extension of treatment time (Fig. 3A). The reactome analysis for differential proteins was enriched in metabolism and cellular response to stress (Fig. 3B). The result revealed once again that metabolism regulation is an important way for cancer cells to respond to drugs. In addition, compared with the control, the whole‐protein rate of change (Rc, Rc = |Log_2_ (Fold change)|) increased with the increasing duration of drug treatment (Fig. S4A, Table S4). There was a significant increase in Rc values when treatment time exceeded 24 h. This is similar to the trend of Rc in the nascent proteome and is consistent with the actual situation (Fig. S4B, Table S5). Notably, a comparison of proteins with the top 15% Rc values in whole proteome and nascent proteome indicates that proteins affected by inhibitors can be found more quickly through nascent proteome analysis (Fig. 3C, Table S6).

**Fig. 4:**
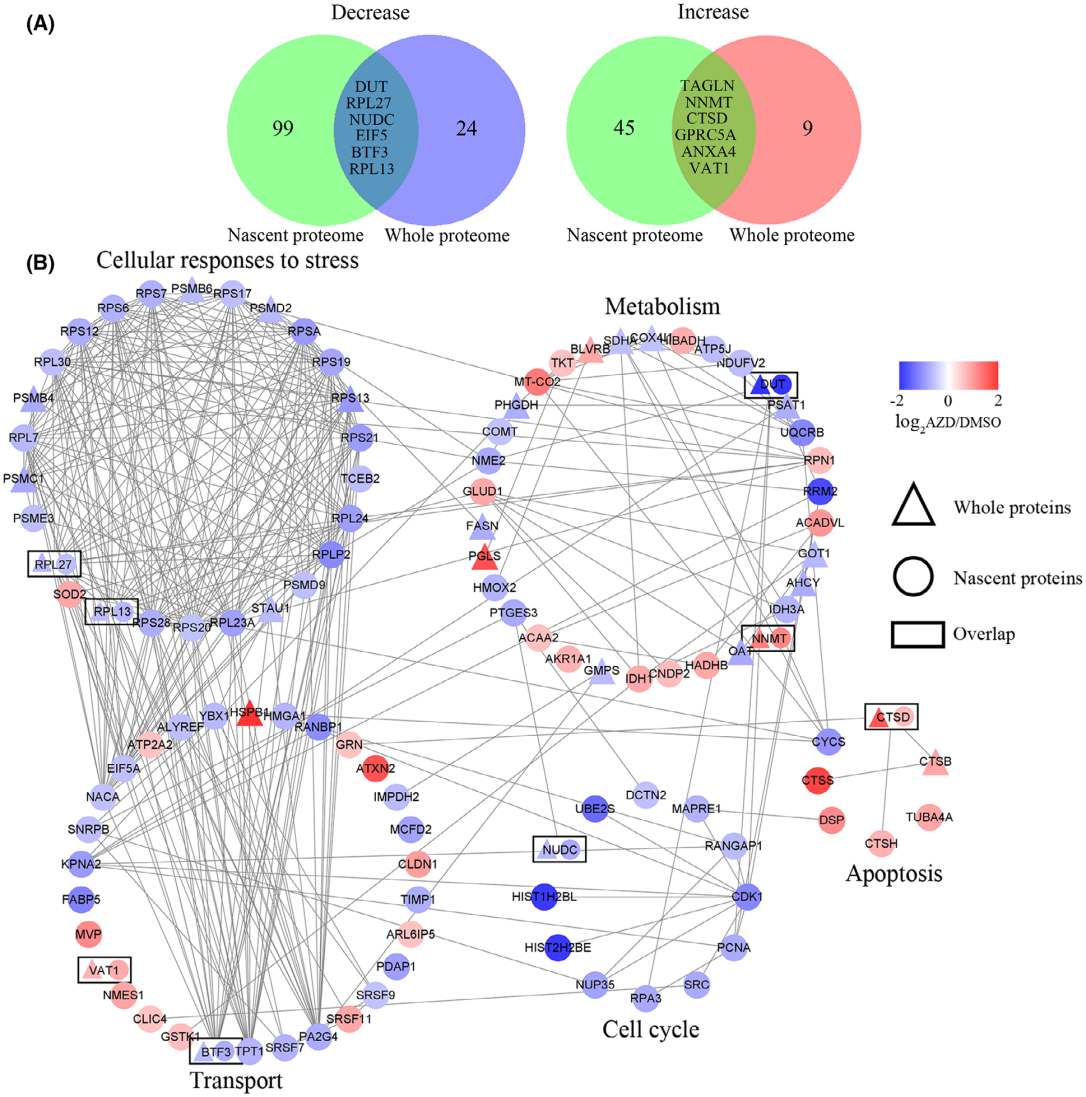
Integration analysis of nascent proteome and whole proteome. (A) Venn diagram showing the number of significantly changed proteins identified in nascent proteome and whole proteome. (B) Reactome pathways enriched for significantly altered proteins in nascent proteome and whole proteome. Only interactions with a STRING score ≥ 0.7 are shown. Node colors are linearly related to fold change, and node shapes represent protein identification method: triangles represent proteins identified via the whole proteome method, circles represent those identified in the nascent proteome, and rectangles represent proteins identified in both methods.

By comparing the whole proteome with the nascent proteome, it was found that the altered proteins were significantly different (Fig. 4A, Table 1). Among them, TAGLN, NNMT, and DUT changed identically in both nascent and whole proteome (Fig. S5). But most of the proteins changed in the nascent and whole proteome were unrelated, which was consistent with a recent study [26]. Therefore, the results indicate that the nascent proteome will provide a new perspective for uncovering the cellular response to inhibitors. To further characterize the significantly altered proteins, we performed a network analysis using string (Fig. 4B). The results revealed the reduced proteins enriched in cellular responses to stress and the cell cycle, indicating that the pathway was inhibited. In contrast, proteins associated with apoptosis increased significantly, suggesting that the inhibitor caused apoptosis. Differential proteins were also enriched in the transport process and metabolism. NNMT was detected in both nascent proteome and whole proteome. NNMT is a metabolizing enzyme and is involved in the biotransformation of many drugs and xenobiotics [27]. NNMT is also a master metabolic regulator of cancer‐associated fibroblasts [28] and could enhance chemoresistance in breast cancer [29]. Clinical correlation analyses revealed that NNMT protein overexpression is significantly associated with cancer stage and shorter overall survival (Fig. S6A,B). So, further investigation of its role in drug treatment is warranted.

**Table 1:** Comparison of proteins changed in nascent proteome and whole proteome in response to AZD9291 treatment as based on the *P* value and fold change.

Protein ID	Protein name	Fold change (Log2R/S)	
		Nascent	Whole
P33316	DUT	−1.98083	−2.3155
P62805	HIST1H4A	−1.64466	−0.0999
P61604	HSPE1	−1.24764	0.061001
P05387	RPLP2	−1.15609	−0.36491
Q9GZZ1	NAA50	−0.98486	−0.55927
Q13442	PDAP1	−0.94054	−0.45046
P62750	RPL23A	−0.92998	−0.13432
Q9Y266	NUDC	−0.83585	−0.6966
P20290	BTF3	−0.79211	−0.66835
P55010	EIF5	−0.78112	−0.68741
P62081	RPS7	−0.77103	0.033941
Q13405	MRPL49	−0.77073	−0.11634
Q15691	MAPRE1	−0.75958	−0.48135
P24534	EEF1B2	−0.7546	−0.2176
P26373	RPL13	−0.69793	−0.65426
P61353	RPL27	−0.64139	−0.71553
O15372	EIF3H	−0.63479	−0.21486
P29401	TKT	0.606781	0.266545
Q9NZM1	MYOF	0.649267	0.161592
Q96KP4	CNDP2	0.667379	0.333596
P07339	CTSD	0.684844	1.65379
Q8NFJ5	GPRC5A	0.714367	1.384119
P31937	HIBADH	0.794381	0.430593
Q99536	VAT1	0.828107	0.731447
O75874	IDH1	0.83426	0.409387
P09525	ANXA4	0.83617	0.796553
P55084	HADHB	0.852893	0.374474
P07099	EPHX1	1.029498	0.195719
Q14764	MVP	1.142525	0.291669
P40261	NNMT	1.183347	1.256367
Q01995	TAGLN	1.362663	1.915405

### 3.4 NNMT is associated with H1975 cells in response to AZD9291

**Fig. 5:**
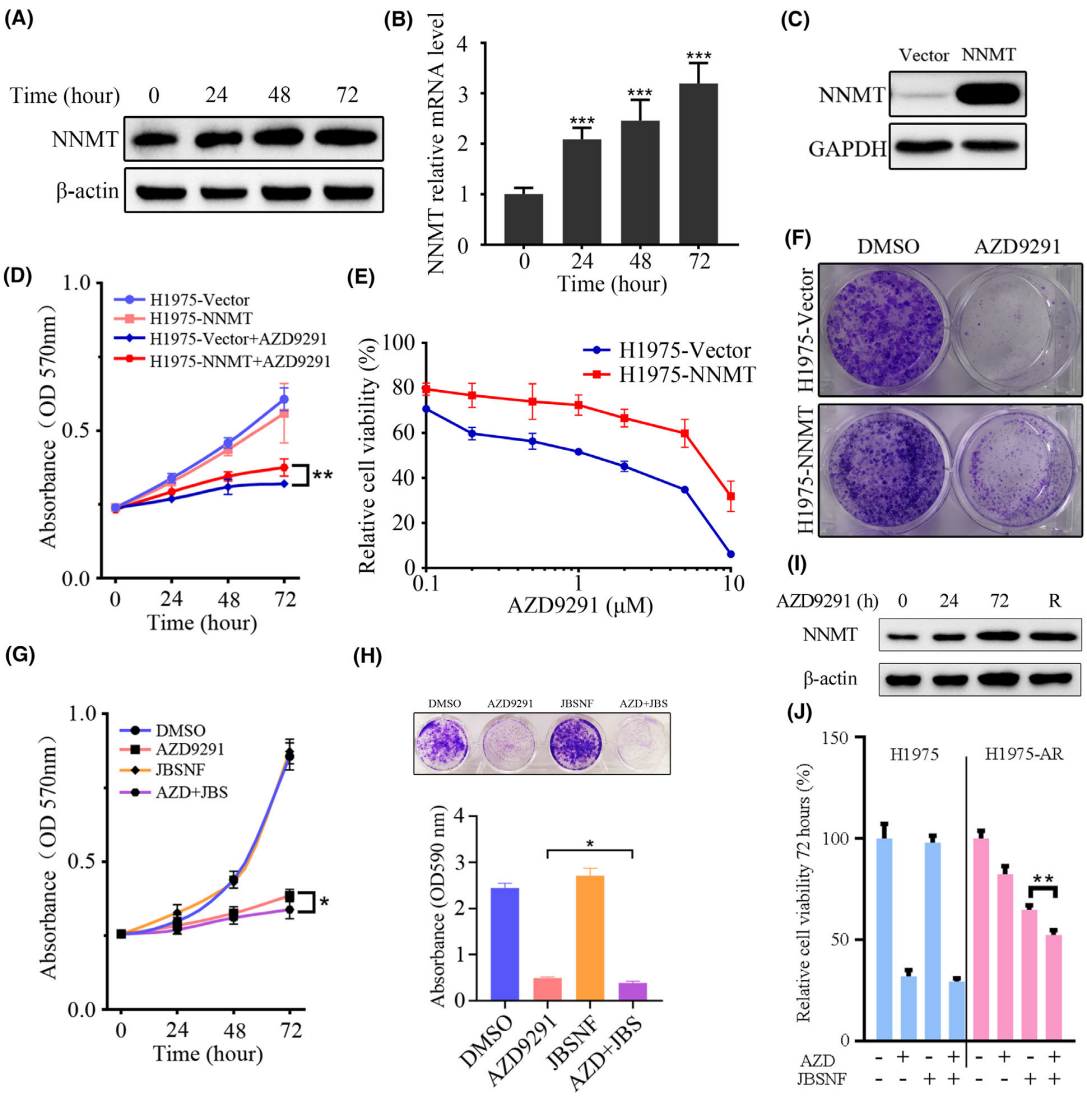
Role of nicotinamide *N*‐methyltransferase (NNMT) in H1975 cell response to AZD9291 (1 μm). (A) WB assay to show the NNMT level after AZD9291 treatment (*n* = 3). (B) The NNMT mRNA levels during AZD9291 treatment were assayed by qPCR (*n* = 4). (C) The expression of NNMT in H1975 was measured after transfection with the NNMT overexpression (NNMT‐OE) vector (*n* = 3). (D) Proliferation analysis of H1975‐vector and H1975‐NNMT cells within 72 h after treatment with AZD9291 were assayed by MTT (*n* = 4). (E) Relative cell viability analysis of H1975‐vector and H1975‐NNMT cells treated with different concentrations of AZD9291 were assayed by MTT (*n* = 4). (F) Colony formation assay for H1975‐vector and H1975‐NNMT cells treated with AZD9291 (*n* = 3). (G) Proliferation analysis of H1975 cells after treatment with AZD9291 or JBSNF‐000088 (JBSNF) (10 μm) were assayed by MTT (*n* = 4). (H) Image and statistics of colony formation assay for H1975 treated with AZD9291 or JBSNF (10 μm) (*n* = 3). (I) WB assay to show the NNMT level after resistance development (*n* = 3). (J) Relative cell viability analysis after AZD9291 or JBSNF (10 μm) treatment (*n* = 4). Error bars depicting standard deviation. Significance determined by Student *t*‐test. *P < 0.05, **P < 0.01, ***P < 0.001.

MS results showed that NNMT was significantly increased after AZD9291 treatment. WB and qPCR results further demonstrated a significant increase of NNMT in protein and transcription levels (Fig. 5A,B). In order to exclude the effect of protein degradation, we used MG132 to inhibit protein degradation during AZD9291 treatment. We compared the relative level of NNMT before and after MG132 treatment. The results indicated that the protein synthesis of NNMT increased under AZD9291 treatment (Fig. S7). To further investigate the effect of NNMT on drug resistance, we stably constructed a cell line by overexpressing NNMT in H1975 cells (H1975‐NNMT) (Fig. 5C). Compared with the control group, we found that AZD9291 had a reduced inhibitory effect on H1975 cells with excess NNMT (Fig. 5D,E). We also performed a colony formation assay to assess the role of NNMT in H1975 cell proliferation. Fewer clones were formed in the H1975‐vector group after AZD9291 treatment, compared to the H1975‐NNMT group (Fig. 5F).

Furthermore, we used JBSNF, an inhibitor of NNMT, combined with AZD9291 to treat H1975 cells. The relative viability of H1975 cells treated with AZD9291 or the combination with JBSNF for 72 h was evaluated by MTT (Fig. 5G). The results showed that the JBSNF could not inhibit the growth of H1975 cells. However, the combination of AZD9291 with JBSNF inhibited cell viability more efficiently in H1975 cells than single inhibitor treatment. More importantly, AZD9291 successfully inhibited the colony formation of H1975 cells, while the combination of AZD9291 with JBSNF exerted the strongest inhibition of colony formation, which was consistent with the cell viability assay (Fig. 5H). The result showed that NNMT was significantly increased in the resistance cells (Fig. 5I). Moreover, H1975R cells with high expression of NNMT were more sensitive to JBSNF than H1975 cells, and AZD9291 combined with JBSNF significantly inhibited the viability of H1975R cells (Fig. 5J). The above results suggested that NNMT overexpression promoted the resistance development and inhibition of NNMT provided a way to overcome drug resistance.

### 3.5 Systematic analysis of phosphoproteome effected by NNMT in H1975 cells

**Fig. 6:**
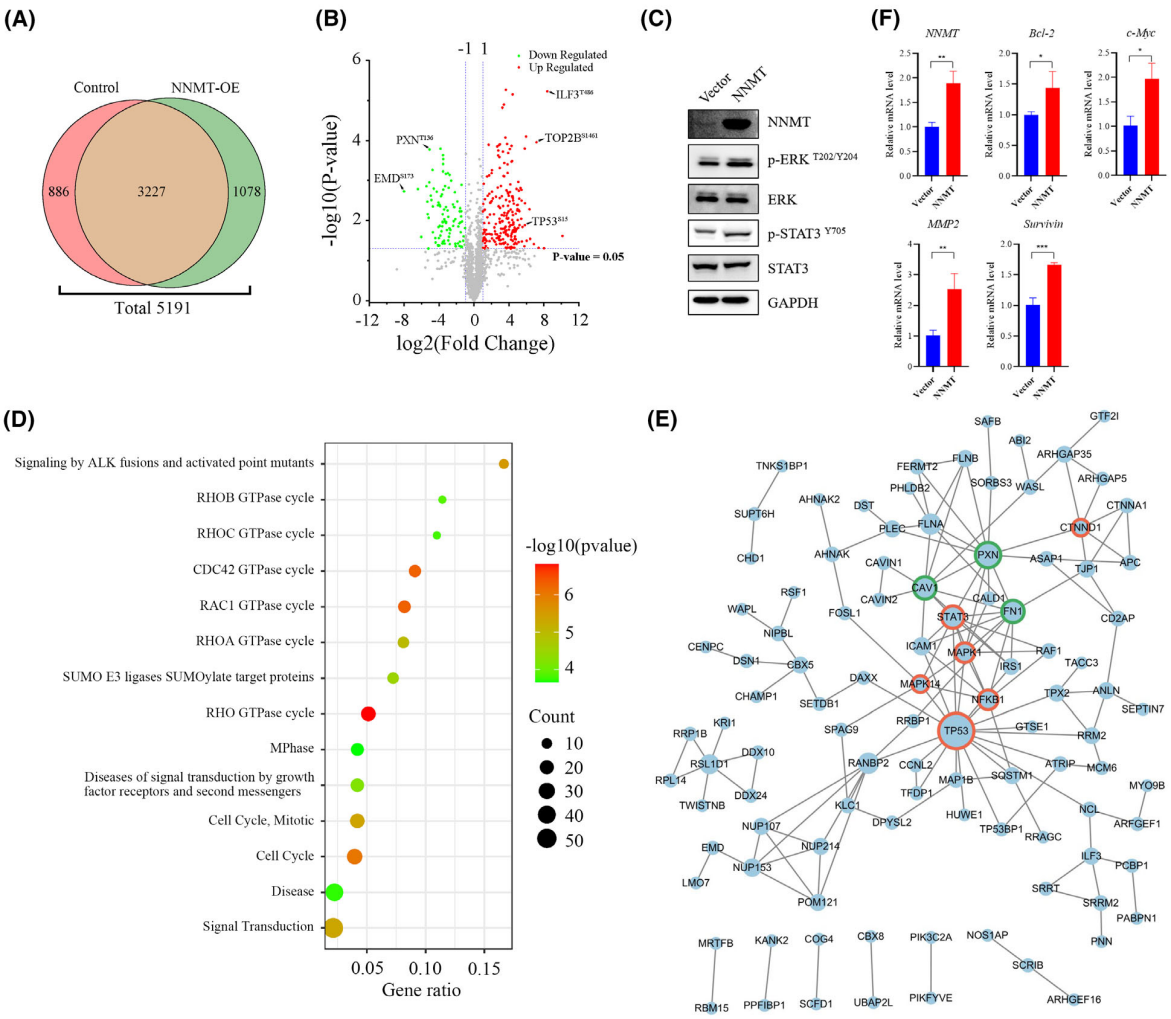
Phosphoproteome analysis of H1975 cells in response to NNMT overexpression. (A) Venn diagram showing the number of phosphorylation sites identified. (B) Volcano plots showing the phosphorylation sites changed after NNMT overexpression (*n* = 2). (C) WB assay for phosphorylation of Erk1/2 and STAT3 after NNMT overexpression (*n* = 3). (D) Reactome analysis for the phosphorylation significantly changed proteins. (E) Protein–protein interaction network for the significantly changed phosphoproteins. Only interactions with a string score ≥ 0.7 are shown. Node size is positively correlated with the number of interacting proteins, and the circle color represents the protein type (red means transcription factor, and green means adhesion protein) (F) qPCR assay for transcription levels of STAT3‐regulated proteins after overexpression of NNMT (*n* = 3). Error bars depicting standard deviation. Significance determined by Student *t*‐test. *P < 0.05, **P < 0.01, ***P < 0.001.

Previous studies have shown that NNMT can affect the methylation and phosphorylation of proteins. Many studies have systematically reported the effect of NNMT on cell stemness by regulating the methylation of histone and DNA. We also found dynamic changes in cell stemness by analyzing transcription factors associated with cell stemness, SOX2 and NANOG (Fig. S8A). However, the effect on phosphorylation has not been systematically investigated and should not be ignored. Therefore, we sought to elucidate the mechanism of NNMT in drug resistance through its effect on the phosphoproteome. We identified a total of 5191 phosphopeptides in H1975 and H1975‐NNMT cells, of which 3227 were present in both groups (Fig. 6A, Table S7). The data quality and reliability were examined by PCA and heatmap analysis (Fig. S8B,C). The 2‐fold change and *P* < 0.05 were selected as the threshold to determine whether a phosphorylation site was affected by NNMT (Fig. 6B). The results showed that 339 phosphopeptides were affected by the overexpression of NNMT, of which 236 phosphopeptides were significantly upregulated and 103 phosphopeptides were downregulated. WB results also showed that the overexpression of NNMT led to increased phosphorylation of transcription factors MAPK1/3 and STAT3 (Fig. 6C). Further reactome analysis revealed that major clusters of proteins with elevated phosphorylation were associated with the Rho GTPases cycle and cell cycle, suggesting that they were influenced by NNMT (Fig. 6D). String analysis revealed that proteins such as MAPK1, TP53, NFKB1, STAT3, and FN1 play key roles in the protein–protein interaction network (Fig. 6E). Previous work [30, 31] has shown that NNMT can affect the phosphorylation of STAT3, which in turn affects tumor resistance. Subsequent analysis showed that NNMT overexpression led to increased transcription of Bcl‐2, c‐Myc, MMP2, and Survivin, whose transcription is regulated by STAT3 (Fig. 6F). Phosphoproteome analysis further showed that the effect of NNMT on drug resistance was related to its effect on the intracellular phosphorylation signals, especially on transcription factors.

## 4 Discussion

Increasing evidence shows that the study of cancer drug resistance should follow the whole process of drug therapy, not only the two stable states of sensitivity and resistance, which is particularly important for elucidating the mechanism of drug resistance and early intervention [11]. Because drug‐induced changes in protein levels are disturbed by a large number of background proteins, it takes longer to detect their changes after drug treatment. However, as the treatment lasts longer, more proteins change, interfering with the detection of key regulatory factors. In this work, we used AHA to label the nascent proteome, which can remove the background interference of a large number of existing proteins, so that proteins that respond to drug therapy can be displayed more sensitively. This is crucial for studying how cancer responds to drugs, which has not been reported previously. Udayan's work showed that TAGLN was significantly increased in drug‐resistant cells [22]. Nascent proteome analysis further demonstrated that TAGLN synthesis was significantly increased within 24 h of drug treatment. GO analysis of nascent proteome showed that the metabolism of heterocyclic or aromatic compounds changed significantly. Notably, AZD9291 contains heterocyclic and aromatic structures, and cancer cells may be able to survive by regulating drug metabolism. Therefore, dysregulation of metabolic signaling is an important feature of tumors, and targeting metabolic regulators may represent an opportunity for overcoming drug resistance.

Furthermore, we performed a whole‐protein level analysis of the 614 significant proteins identified in the nascent proteome by scheduled PRM, a targeted quantitation strategy independent of antibodies. By comparing the whole proteome with the nascent proteome, we observed few proteins exhibiting similar change trends, underscoring the complementarity of nascent proteome analysis to conventional whole‐protein analysis. Notably, no proteins exhibited opposite change trends between the two datasets, suggesting some degree of similarity. Cluster analysis revealed that the reduced proteins were enriched in cellular responses to stress and cell cycle following AZD9291 treatment, indicating that the pathway was inhibited. Conversely, increased proteins in the apoptotic pathway indicated enhanced apoptosis. Differential proteins were also enriched in transport processes and metabolism, and previous work has shown that changes in these two pathways are associated with drug resistance [32]. Notably, changes in metabolism‐related proteins such as NNMT, PGLS, MT‐CO2, and DUT highlight the role of metabolic reprogramming in tumor resistance, providing new strategies for resistance therapy.

NNMT, a metabolic enzyme, plays a crucial role in therapeutic response. Previous studies have found that NNMT promotes tumor development and drug resistance and is associated with its effects on histone and DNA methylation. We also found that NNMT overexpression conferred H1975 cells resistance to EGFR inhibitor, while its inhibition enhanced drug efficacy, particularly in resistant cells. NNMT promotes cancer resistance to kinase inhibitors, which must be related to phosphorylation signals. However, the effect of NNMT on phosphorylation has not been systematically studied. In this study, we reveal for the first time the impact of NNMT on phosphorylation networks. Our studies have shown that NNMT affects the phosphorylation of numerous transcription factors, and these proteins are involved in cellular processes, including cell cycle and Rho GTPases cycle. The latest work shows that NNMT can promote the cell cycle of tumor cells [33]. Moreover, the Rho GTPases regulate basic biological processes such as cell locomotion, cell division, and morphogenesis [34]. The effect of NNMT on cell morphology reported in previous work may be related to Rho GTPases. Our study suggested that targeting NNMT can be a novel strategy to intervene in the development of or overcome tumor drug resistance in cancer therapy.

## 5 Conclusions

In summary, we adopted a workflow including metabolic labeling, TAD‐resin enrichment, and MS quantification for comprehensively understanding the changes of nascent proteins at the initial stage of drug treatment. We identified a comprehensive dataset of nascent proteins at 4, 8, and 24 h of AZD9291 treatment. Our results underscored the enrichment of differential proteins in metabolism‐related pathways, implicating metabolic alterations in EGFR inhibition. We demonstrated that NNMT upregulation promoted resistance to AZD9291, while NNMT inhibition enhanced drug efficacy, particularly in resistant cells. Additionally, quantification of whole proteins by PRM deepened protein analysis compared to nascent proteins. Furthermore, the results revealed the effect of NNMT on transcription factor phosphorylation, particularly in the Rho GTPases cycle and cell cycle. Together, our investigation of nascent proteome alterations provided new insights into the immediate responses of cancer cells to AZD9291 treatment, highlighting NNMT as a potential therapeutic target.

## Conflict of interest

The authors declare no conflict of interest.

## Author contributions

ZH and ZW conducted the experiments, analyzed the results, and wrote the first draft. FY and XH provided the guidance for solving the problems in the process of experiments. LL assisted in analyzing the data in this study. HL conceived and designed the study and revised the manuscript. All authors read and approved the final manuscript.

## Supporting information


**Fig. S1.** Changes in H1975 cells after tyrosine kinase inhibitor treatment.
**Fig. S2.** Nascent proteome analyzed by LC–MS/MS after AZD9291 treatment.
**Fig. S3.** Chromatography of selected unique peptides analysis by LC‐PRM.
**Fig. S4.** Rc value of the proteome in each group after different treatment time.
**Fig. S5.** The quantitative results of PRM and LFQ for TAGLN, NNMT and DUT.
**Fig. S6.** Effect of NNMT overexpression on cancer progression.
**Fig. S7.** Analysis of NNMT synthesis levels in H1975 cells following AZD9291 treatment.
**Fig. S8.** The effects of NNMT on stemness and phosphorylation of H1975 cells.

**Table S1.** Nascent proteins that identified in H1975 cells after treated by AZD9291.
**Table S2.** List of unique peptides used for schedule‐PRM.
**Table S3.** Quantification results of unique peptides by schedule‐PRM.
**Table S4.** Rc value for whole proteome.
**Table S5.** Rc value for nascent proteome.
**Table S6.** The proteins with the top 15% of Rc value in nascent or whole proteins.
**Table S7.** Phosphoproteome analysis of H1975 cells in response to NNMT overexpression.

## Data Availability

The mass spectrometry proteomics data have been deposited to the ProteomeXchange Consortium (http://proteomecentral.proteomexchange.org) via the iProX partner repository with the dataset identifier PXD057767. All other data needed to evaluate the conclusions in the paper are present in the article or the Supporting Information.
