# Supplementary material for: Nicotinamide N‐methyltransferase promotes drug resistance in lung cancer, as revealed by nascent proteomic profiling
Source: Mol Oncol. 2025 Jul 18;20(2):541–54. doi: 10.1002/1878-0261.70097 (PMC12936426; doi:10.1002/1878-0261.70097)
Supplement: Supplementary file 1 — Fig. S1. Changes in H1975 cells after tyrosine kinase inhibitor treatment. Fig. S2. Nascent proteome analyzed by LC–MS/MS after AZD9291 treatment. Fig. S3. Chromatography of selected unique peptides analysis by LC‐PRM. Fig. S4. Rc value of the proteome in each group after different treatment time. Fig. S5. The quantitative results of PRM and LFQ for TAGLN, NNMT and DUT. Fig. S6. Effect of NNMT overexpression on cancer progression. Fig. S7. Analysis of NNMT synthesis levels in H1975 cells following AZD9291 treatment. Fig. S8. The effects of NNMT on stemness and phosphorylation of H1975 cells. [file MOL2-20-541-s002.docx]

Supporting Information

**Nicotinamide N-Methyltransferase promotes drug resistance in lung cancer, as revealed by nascent proteomic profiling**

Zhanwu Hou^1#^, Zhen Wang^2#^, Fei Yang^1^, Xiao Han^1^, Lei Li^2^, Huadong Liu^2*^

^1^ Ministry of Education (MoE) Key Laboratory of Biomedical Information Engineering, School of Life Science and Technology, Xi'an Jiaotong University, Xi'an, 710049, China;

^2^ School of Life Science and health, University of Health and Rehabilitation Sciences, Qingdao, 266113, China.

^#^ These authors contributed equally to this work.

^*^ Correspondence

Huadong Liu, School of Life Science and health, University of Health and Rehabilitation Sciences, Qingdao, 266113, China; E-mail: huadongliu@uhrs.edu.cn.

**Table of Contents**

**Supporting tables**

**Table S1.** Nascent proteins that identified in H1975 cells after treated by AZD9291.

**Table S2.** List of unique peptides used for schedule-PRM.

**Table S3.** Quantification results of unique peptides by schedule-PRM.

**Table S4.** Rc value for whole proteome.

**Table S5.** Rc value for nascent proteome.

**Table S6.** The proteins with the top 15% of Rc value in nascent or whole proteins.

**Table S7.** Phosphoproteome analysis of H1975 cells in response to NNMT overexpression.

**Supporting Figures**

**Fig. S1.** Changes in H1975 cells after tyrosine kinase inhibitor treatment.

**Fig. S2.** Nascent proteome analyzed by LC-MS/MS after AZD9291 treatment.

**Fig. S3**. Chromatography of selected unique peptides analysis by LC-PRM.

**Fig. S4**. Rc value of the proteome in each group after different treatment time.

**Fig. S5.** The quantitative results of PRM and LFQ for TAGLN, NNMT and DUT.

**Fig. S6.** Effect of NNMT overexpression on cancer progression.

**Fig. S7.** Analysis of NNMT synthesis levels in H1975 cells following AZD9291 treatment.

**Fig. S8.** The effects of NNMT on stemness and phosphorylation of H1975 cells.

**Supporting Figures**


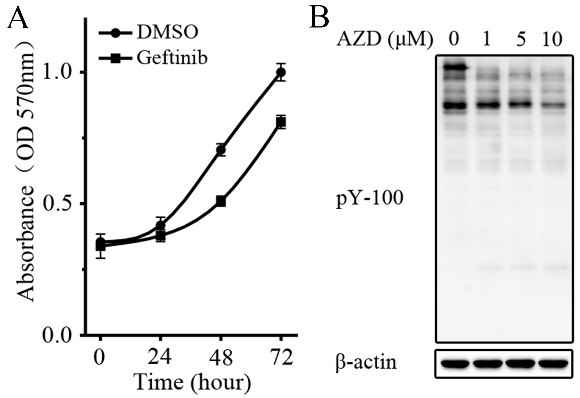


**Fig. S1.** Changes in H1975 cells after tyrosine kinase inhibitor treatment. (A) Proliferation of H1975 cells treated with 1 μM geftinib (n=6). Error bars indicate standard deviation. (B) Western blot analysis of the phospho-tyrosine proteome after AZD9291 (AZD) treatment (n=3).


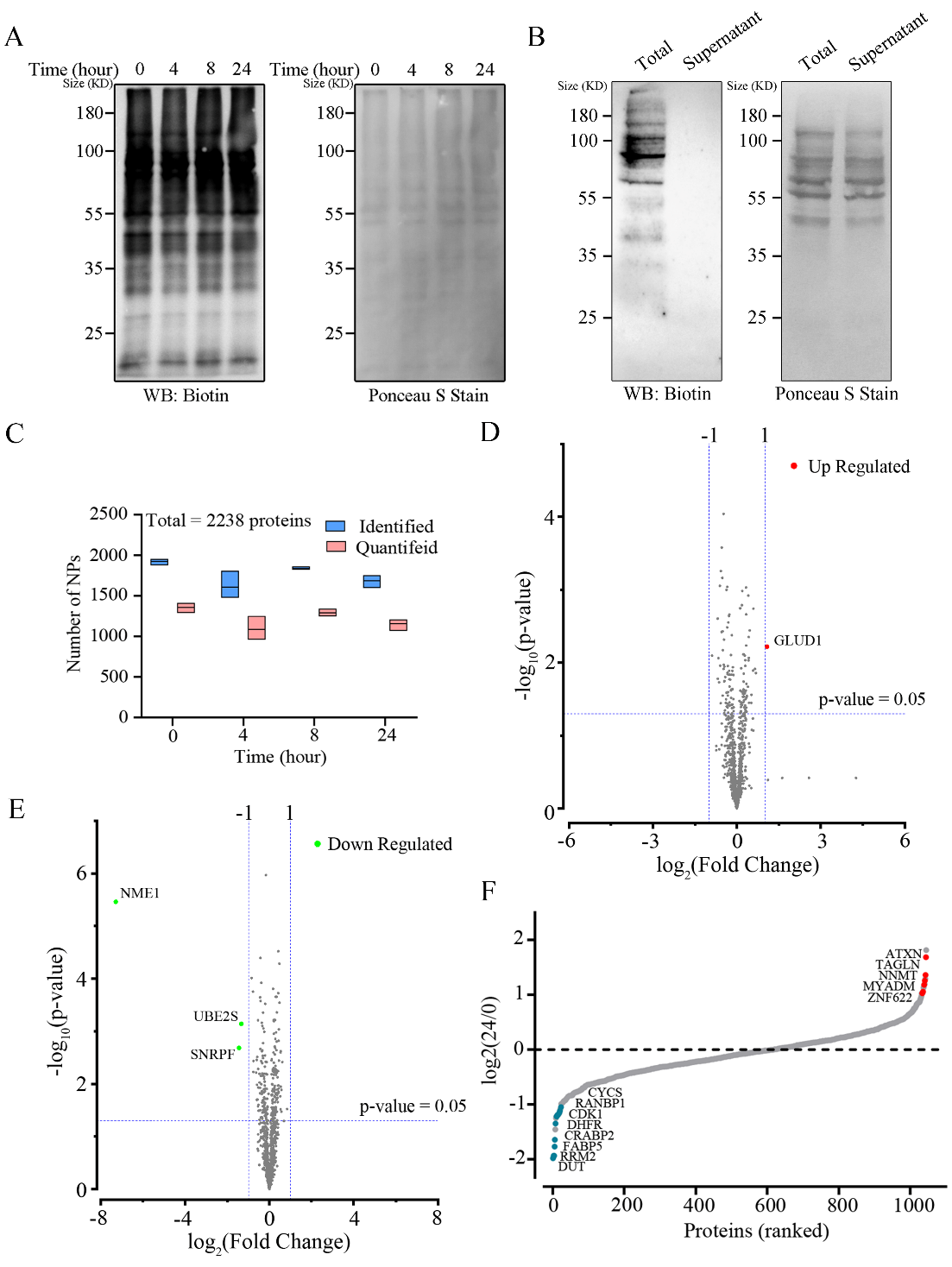


**Fig. S2.** Nascent proteome analyzed by LC-MS/MS after AZD9291 (1 μM) treatment. (A) The nascent proteome of AZD9291 treated for different time was analyzed by WB (n=3). (B) Nascent protein enriched success by TAD-resin (n=3). (C) Nascent proteins that identified by LC-MS/MS (n=3). Volcano plots showing the nascent proteins changed (n=3) after AZD9291-treated for 4 hours (D) or 8 hours (E). (F) Rank plot visualization of nascent proteins in AZD9291 treated versus control (n=3).


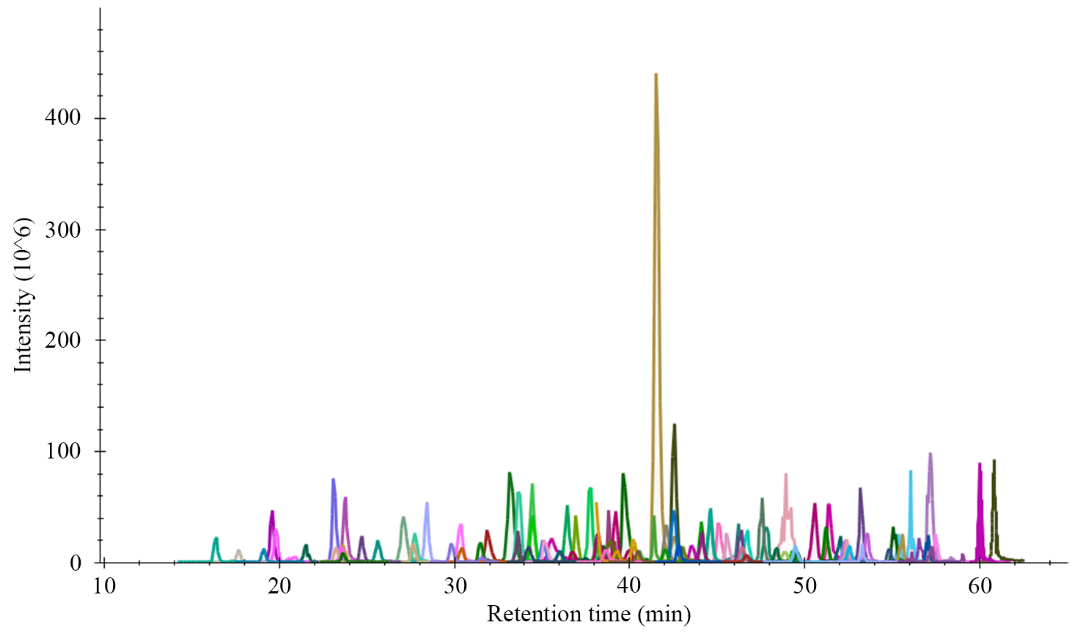


**Fig. S3**. Chromatography of selected unique peptides analysis by LC-PRM.


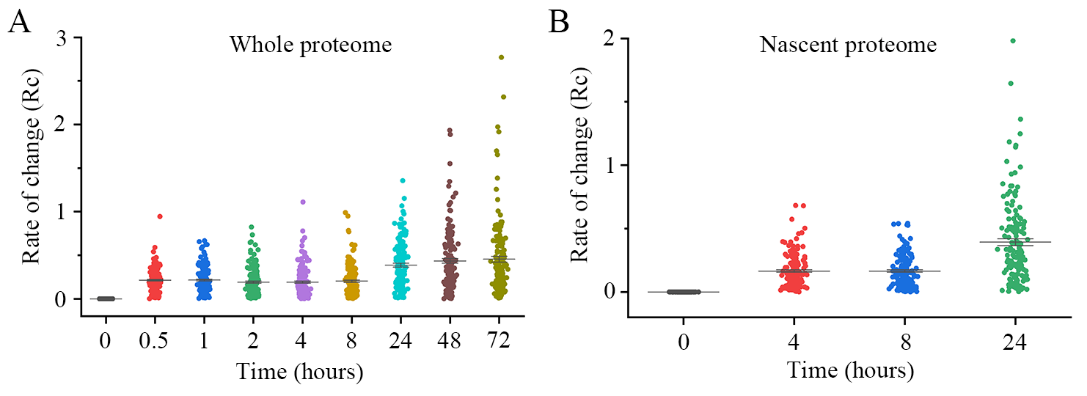


**Fig. S4**. Rc value of the proteome in each group after different treatment time. (A) Whole proteome level. (B) Nascent proteome level.


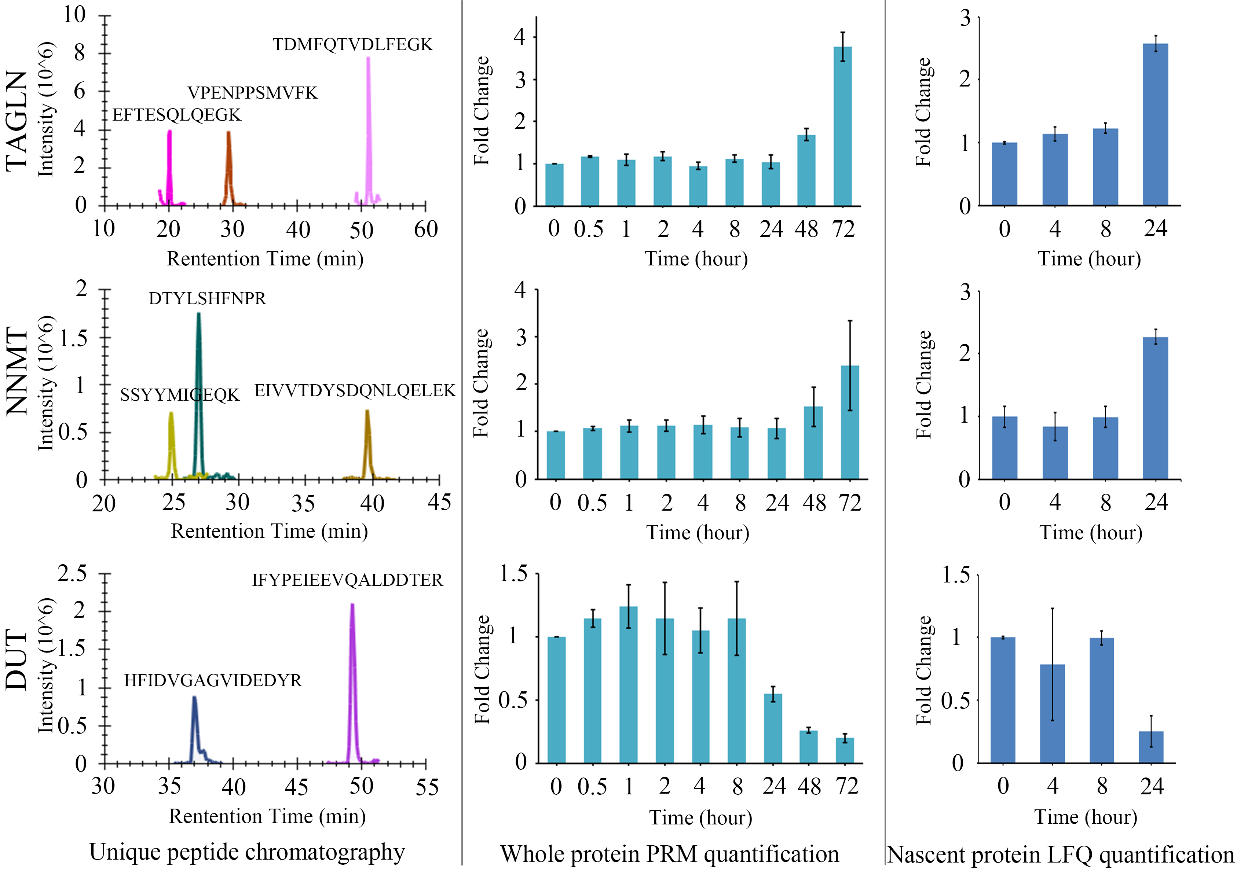


**Fig. S5.** The quantitative results of PRM (n=3) and LFQ (n=3) for TAGLN, NNMT and DUT. Error bars indicate standard deviation.


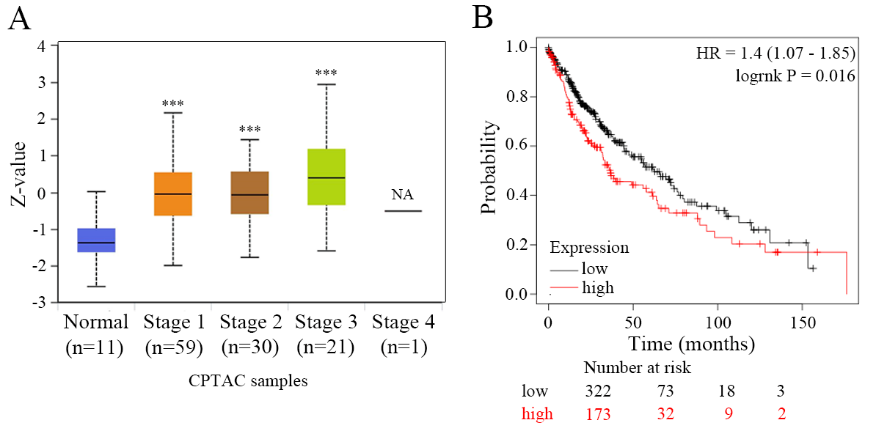


**Fig. S6.** Effect of NNMT overexpression on lung adenocarcinoma progression. (A) Expression of NNMT was correlated with cancer stage using data from the Clinical Proteomic Tumor Analysis Consortium (CPTAC) database. Z-values represent standard deviations from the median across samples for the given cancer type. Error bars depicting standard deviation. Signiﬁcance determined by Student t-test (Normal vs Stage 1/2/3/4). ****P* < 0.001. NA means Not Available. (B) The correlation between NNMT expression and patients' overall survival was analyzed using The Cancer Genome Atlas (TCGA) dataset, with Kaplan–Meier survival curves and log–rank test performed to assess the association. Patients were stratified into high and low NNMT expression groups based on median expression values.


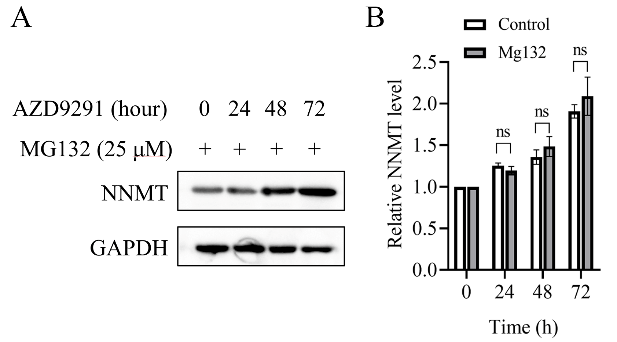


**Fig. S7.** Analysis of NNMT synthesis levels in H1975 cells following AZD9291 (1μM) treatment. (A) WB assay showed the NNMT level after incubation with AZD9291 and MG132 (n = 3). (B) WB (A) densitometric analysis was performed to compare the relative level of NNMT before and after MG132 (25 μM) treatment in the presence of AZD9291 (n = 3). Error bars indicate standard deviation. ns, no significant.


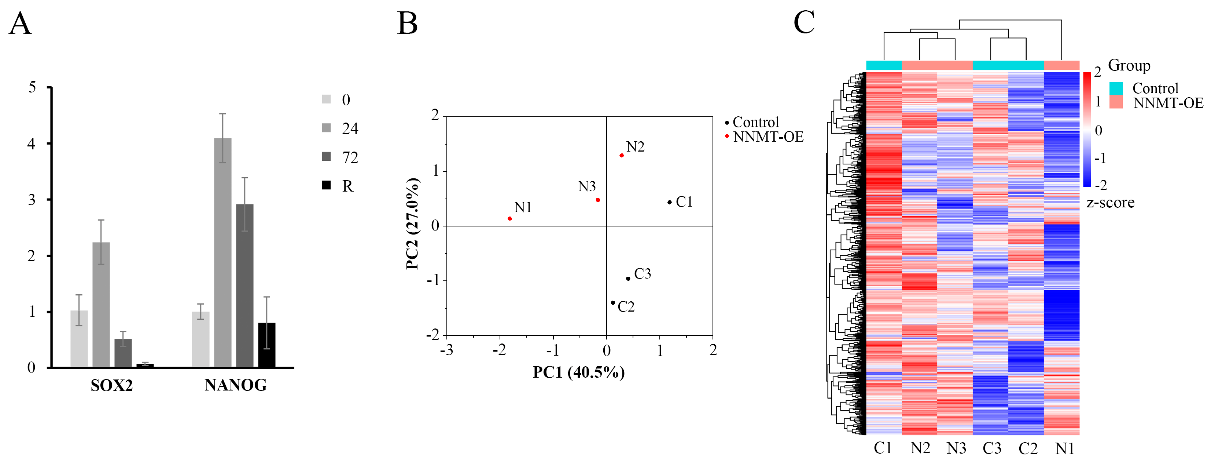


**Fig. S8.** The effects of NNMT on stemness and phosphorylation of H1975 cells. (A) qPCR assay for transcription levels of SOX2 and NANOG (n=3) after treated with 1 μM AZD9291. Error bars indicate standard deviation. Phosphoproteome of H1975 cells in response to NNMT overexpression (n=3) were analyzed by PCA (B) and heatmap (C).
